# Unveiling directional physiological coupling in human-horse interactions

**DOI:** 10.1016/j.isci.2024.110857

**Published:** 2024-08-31

**Authors:** Alejandro Luis Callara, Chiara Scopa, Laura Contalbrigo, Antonio Lanatà, Enzo Pasquale Scilingo, Paolo Baragli, Alberto Greco

**Affiliations:** 1 Department of Information Engineering, University of Pisa, Via G. Caruso 16, Pisa, 56122 Pisa, Italy; 2 Research Center “E. Piaggio”, Largo Lucio Lazzarino 1, Pisa, 56122 Pisa, Italy; 3 Department of Medicine and Surgery, Unit of Neuroscience, University of Parma, Viale delle Scienze 11/A, Parma, 43124 Parma, Italy; 4 National Reference Centre for Animal Assisted Interventions, Istituto Zooprofilattico Sperimentale delle Venezie,Viale dell’Università 10, 35020 Legnaro, Italy; 5 Department of Information Engineering, University of Florence, Via di Santa Marta 3, Firenze, 50139 Firenze, Italy; 6 Department of Veterinary Sciences, University of Pisa, Viale delle Piagge 2, Pisa, 56124 Pisa, Italy

**Keywords:** Equine behavior, Zoology, Behavioral neuroscience

## Abstract

This research investigates the human-horse bond, aiming to unveil the physiological mechanisms regulating interspecies interactions. We hypothesized observing a physiological synchronization in human-horse dynamics, akin to human interactions. Through time-frequency Granger causality analysis of heart rate variability (HRV) and behavioral data, this study reveals the establishment of bidirectional synchronization in HRV between humans and horses. The coupling directionality is influenced by behavior and familiarity. In exploration scenarios led by horses, bidirectional interactions occur, particularly with familiar individuals. Conversely, during human-led activities such as grooming, physiological connectivity direction varies based on the familiarity level. In addition, the methodology allows in-depth analysis of sympathetic and parasympathetic nervous system contributions, highlighting their intricate role in the human-horse relationship. Such a physiological coupling estimate, correlated with behavioral data, provides a quantitative tool applicable across contexts and species This holds significant promise for assessing animal-assisted therapies and for applications in sports and various animal-related domains.

## Highlights

• Demonstrated bidirectional HRV synchronization between humans and horses
• Causality of physiological coupling varies by familiarity and interaction type
• Physiological synchronization is linked to specific behavioral dynamics
• Broad applications in animal-assisted therapy and human-animal interaction studies

## Graphical abstract

**Figure:**
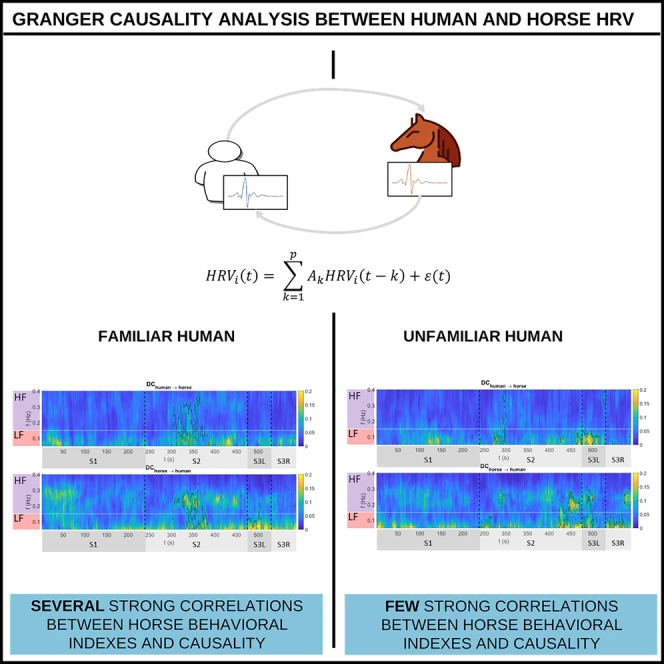

## Introduction

Interactions with animals have long fascinated researchers across a range of scientific disciplines, offering unique opportunities to explore the intricacies of communication, bonding, and shared experiences. Particularly, the human-horse bond holds a special place because of its historical and practical significance. Horses have been our companions, helpers, and sources of inspiration for centuries, playing a vital role in many aspects of human life, from transport and agriculture to sport and, more recently, therapy.^1^ This deep-seated bond is no mere coincidence but it can be attributed to the horses’ capacity to perceive human behaviors and emotional states,^2^ as first noted in the famous case of Clever Hans^3^ and to their advanced socio-cognitive abilities, which leads to a complex multilevel social organization.^4^ These abilities include the cross-modal recognition of conspecifics,^5^ reconciliatory interactions,^6^ conspecifics’ facial change recognition,^7^ and emotional communication through vocalizations.^8^

Measuring human-horse interaction extends beyond simple academic curiosity, it directly impacts the welfare of horses, the safety of humans, and the efficacy of training methods, and plays a pivotal role in therapeutic settings where horses serve as effective healers, offering comfort and companionship to people suffering from intellectual disabilities or physical impairments.^9^^,^^10^ It also contributes to our broader understanding of animal behavior, cognition, and emotion, enriching our knowledge of the natural world.

The study of human-animal interplay has traditionally focused on behavioral observations and vocalizations.^11^ However, such a narrow focus could hinder a comprehensive understanding of this phenomenon. Indeed, much such as human interactions, inter-species interplay could occur also at the physiological level. Notably, in humans, there is neurophysiological evidence that social interactions are associated with synchronized brain activity^12^ and oscillatory coupling of other biological functions, such as respiration, electrodermal,^13^ and cardiac activity.^14^ Particularly, the autonomic nervous system (ANS) is at the core of these interactions and synchronization.^15^ This is not unexpected given ANS’s pivotal role in psychophysiological regulation in humans but also animals, modulating their emotions and interactions and serving as the underlying mechanism for these complex dynamics. One valuable correlate of ANS activity is heart rate variability (HRV), which reflects the fluctuation in the time interval between consecutive heartbeats. HRV has been extensively studied in both humans and horses and has proven to be a robust tool for assessing autonomic function, emotional regulation, and overall well-being.^15^^,^^16^ Over the past years, there has been a surge of interest in comparing HRV patterns and investigating potential correlations across subjects and different species.^17^^,^^18^^,^^19^^,^^20^

Since the aforementioned physiological synchronization and the related coordinated human social behaviors are socially adaptive and result in a stronger linkage between the members of a group or a dyad,^21^ it is reasonable to assume that they may not be confined to humans^22^ but shared with other social animal species. Specifically, horse-human coexistence dates back to the end of the third millennium BC ^23^ having granted all the time needed to sharpen communication and ensure beneficial interactions. Since the implied adaptive value of synchronization, in this research, we expect to find a coupling between the physiological signals of humans and horses while interacting.

To explore this hypothesis, we have examined the causal interaction between the HRV time series of humans and horses interacting under various experimental conditions. Particularly, we employed a measure of Granger Causality to assess the causal relationship between two time series. By applying this method to HRV data, we can gain a deeper understanding of how one organism’s physiological state may influence the other’s. This could potentially reveal bidirectional causal physiological links during specific interactions between humans and horses.

To elicit varying degrees of interaction between the human and horse participants we designed an experimental protocol, which involved three distinct sessions. Initially, the human and horse were separated, allowing for baseline measurements of HRV. Subsequently, the horse was allowed to explore the stationary human, establishing a dynamic environment for potential interspecies bonding. Finally, the human groomed the horse on both sides, further enhancing the physical and emotional connection between the individuals. We recorded simultaneous ECGs from twenty horses interacting with twenty pairs of humans. Within each pair, the individuals exhibited two distinct levels of familiarity with the horse they were interacting with - one individual had a low level of familiarity, while the other had a high level.^24^ Indeed, as it is easier for two human subjects to synchronize their signals when they are familiar with each other,^25^^,^^26^^,^^27^ familiarity can also play a significant role in human-animal interactions^24^^,^^28^ and influence equine behaviors.^29^

**Figure 1:**
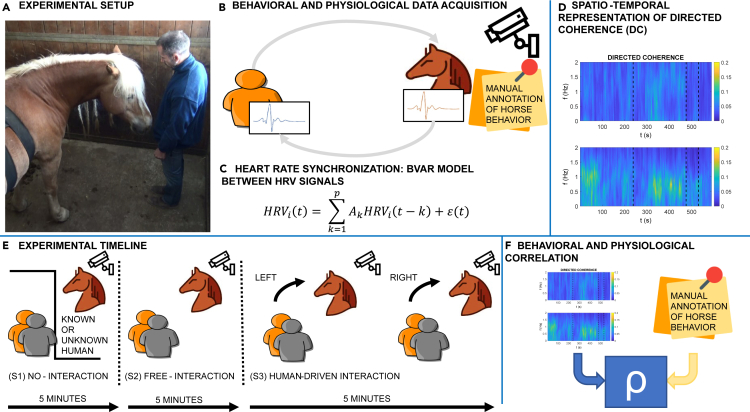
Framework of the study (A) Experimental setup. (B) Behavioral and physiological data were acquired during the whole experiment. Behavioral data was obtained by analyzing videos of the experiment and by manually continuously annotating explorative behavior, attentive behavior, and indicators of frustration. Physiological data was acquired in terms of ECG recordings from the human and the horse and analyzed with Kubios to extract the HRV signal. (C) HRV from the human and the horse were used to construct time-varying bivariate MVAR models. (D) The directed coherence (DC) was estimated as an index of interaction between the human and the horse. (E) The experiment consisted of 4 different sessions during which the horse and the human experienced different levels of interaction (i.e., no-interaction, free-interaction, human-driven interaction). (F) Behavioral and physiological measures were analyzed using Pearson’s correlation coefficient.

To complement the Granger causality analysis, we incorporated video inspection as a standard method to capture and interpret behavioral signals during each session. This quantitative approach allowed us to associate a meaningful context with the observed HRV patterns and identify potential correlations between physiological and behavioral responses. In particular, we focalized on those behaviors known to be correlated with frustration or motivational conflict in previous studies (see,^30^ for an extensive review) to define how, familiar or unfamiliar people interact or not with horses, were perceived. A summary of our experimental procedure is reported in Figure 1.

The results of this study support the hypothesis that horse-human interaction generates and modulates physiological synchronization and presents a valuable tool to quantify the causal coupling between ANS correlates of sympathovagal activity given by the HRV signals. Also, we corroborate physiological parameters with a detailed frame-by-frame video codification of horses’ behaviors, in terms of frustration acts, attention, and exploration of the environment and/or the human subjects. Overall, this research contributes to the growing body of knowledge on the complexities of interspecies relationships and has implications for multiple scientific disciplines and Animal-Assisted Interventions (AAI) as well.

## Results

### Behavioral measures analysis

Through video inspection, we analyzed possible behavioral differences in horses among experimental conditions. More specifically, based on the selected ethogram, we analyzed the frequency of frustration behaviors (***fru***), frequency and duration of explorative behavior toward the environment (***envexp***) and the human subjects (***humexp***), and frequency and duration of attentive behaviors toward the environment (***envatt***) and the humans subject (***humatt***) (See details in Table 1). To this aim the videos were segmented in time windows of 30s for all experimental conditions (for a total of 30 time windows). Considering the frequency of the selected behaviors, the results of the ANOVA revealed statistically significant differences exclusively among the sessions across all examined features (S1, no interaction, S2, free interaction, S3, human-driven interaction), except for frustration behavior (***fru***), which did not show any significant difference among the experimental conditions. A summary of the ANOVA is reported in Table 2. This finding suggests the prominent role of the type of interaction in influencing behavioral responses, regardless of the familiarity level. To gain a more comprehensive understanding of these findings, we conducted a post-hoc analysis utilizing the Simple Effect Analysis method.

**Table 1:** Description of the behavioral patterns collected during the video-analysis

	Behaviors	Operational definitions
Explorative behavior^31^	Exploring the person (***humexp***), Exploring the environment (***envexp***)	The horse sniffs or licks the human subject or something in the environment, using one of the nostrils or the muzzle or the mouth. Exploring the person can also include pushing, attempting to bite/bite, or nipping.
Attentive behavior^31^	Attention toward the person (***humatt***), Attention toward the environment (***envatt***)	The horse actively directs its attention toward the person or the environment. Eyes and ears are considered good indicators of the direction of attention, and both the monocular and binocular visual fields are evaluated. Attention toward the environment also includes looking out the window of the box.
Indicators of frustration^32^ (***fru***)	vacuum chewing, box walking, exploring band, yawning, scratching, kicking, defecate, head tossing, head/body shaking, whining, non-vocal signals, exhalation, stomping	The horse performs many kinds of coping, repetitive actions to manage a stressful situation or discomfort. These may include continuously chewing without anything in the mouth, persistently walking along the perimeter of the box, eliminative behavior, vocal and non-vocal sounds, and substitution behaviors. Also, any attempt to produce noise or to open the gate of the box.

**Table 2:** Summary of ANOVA results for all features

Effect	Feature	Sum Sq	NumDF	F value	Pr(> F)
Session	***humexp***	1121.28	2	51.5067	1.229e-15 ∗∗∗
	***envexp***	64.579	2	7.6546	0.000851 ∗∗∗
	***humatt***	244.421	2	28.7060	2.275e-10 ∗∗∗
	***envatt***	591.44	2	47.4550	8.467e-15 ∗∗∗
	***fru***	32.965	2	1.0501	0.3542
Familiarity	***humexp***	2.84	1	0.2611	0.6106
	***envexp***	0.079	1	0.0187	0.891491
	***humatt***	0.035	1	0.0082	0.9279
	***envatt***	10.14	1	1.6273	0.2054
	***fru***	15.474	1	0.9858	0.3234
session:familiarity	***humexp***	2.58	2	0.1185	0.8884
	***envexp***	17.526	2	2.0774	0.131220
	***humatt***	6.386	2	0.7500	0.4753
	***envatt***	0.91	2	0.0732	0.9295
	***fru***	8.579	2	0.2733	0.7615

Note: ∗∗∗ indicates *p*-values that are significant and below 0.001.

### Explorative and attentive behavior

The results of the Simple Effect Analysis are reported in Table 3. In the case of ***humexp*** feature, the S2 session stood out significantly from the S3 condition, across both levels of familiarity (with familiar humans, contrast S2 vs. S3 t=−4.868, p<.0001; with unfamiliar humans, t=−5.409, p<.0001). This suggests that the context of the session, where the horse had the freedom to inspect a stationary person within the stall, significantly influenced its attitude in the exploration of the person. During S1, no humans were admitted in the box, therefore the comparison with other experimental conditions regarding ***humexp*** is a foregone conclusion. As expected, for both levels of familiarity, the frequency of the exploration of human subjects is significantly higher in S2 compared to S1. However, it is interesting to note that the frequency of ***humexp*** is so low during S3 that it does not significantly differ from the S1 condition, nor with familiar (t=1.721, p=0.2030) nor with the unfamiliar person (t=1.819, p=0.1691). Regarding the ***envexp***, even though no difference has been found when comparing the frequency of the considered behavior in S3 vs. S2, neither with familiar (t=−2.133, p=0.0890) nor with the unfamiliar person (t=0.237, p=0.9695), our analysis showed distinct patterns of significance when considering the resting session S1. When a familiar person was present in the area, tested horses explored more the environment when the interaction was human-driven (p=0.0286) compared to when they managed the interaction (p=0.8837). When a stranger was in the box on the other hand, horses significantly reduced the exploration of the environment, regardless of the kind of interaction (i.e., in both S2 and S3) compared to when there is no human subject to inspect (p=0.0077 for S2 vs. S1, p=0.0151 for S3 vs. S1).

**Table 3:** Post-hoc simple effect analysis for the feature showing a significant ”Session” Effect

Feature	Familiarity	Contrast	Estimate	df	t-ratio	*p*-value
*humexp*	Familiar	Forced - Rest	1.84	90	1.721	0.2030
		Forced - Voluntary	−5.21	90	−4.868	<**.0001**
		Rest - Voluntary	−7.05	90	−6.589	<**.0001**
	Unfamiliar	Forced - Rest	1.95	90	1.819	0.1691
		Forced - Voluntary	−5.79	90	−5.409	<**.0001**
		Rest - Voluntary	−7.74	90	−7.228	<**.0001**
*envexp*	Familiar	Forced - Rest	−1.737	90	−2.606	**0.0286**
		Forced - Voluntary	−1.421	90	−2.133	0.0890
		Rest - Voluntary	0.316	90	0.474	0.8837
	Unfamiliar	Forced - Rest	−1.895	90	−2.843	**0.0151**
		Forced - Voluntary	0.158	90	0.237	0.9695
		Rest - Voluntary	2.053	90	3.080	**0.0077**
*humatt*	Familiar	Forced - Rest	3.632	90	5.425	<**.0001**
		Forced - Voluntary	1.053	90	1.572	0.2628
		Rest - Voluntary	−2.579	90	−3.852	**0.0006**
	Unfamiliar	Forced - Rest	3.000	90	4.481	**0.0001**
		Forced - Voluntary	−0.105	90	−0.157	0.9865
		Rest - Voluntary	−3.105	90	−4.639	<**.0001**
*envatt*	Familiar	Forced - Rest	−5.16	90	−6.368	<**.0001**
		Forced - Voluntary	−1.32	90	−1.625	0.2406
		Rest - Voluntary	3.84	90	4.744	<**.0001**
	Unfamiliar	Forced - Rest	−5.58	90	−6.888	<**.0001**
		Forced - Voluntary	−1.42	90	−1.755	0.1909
		Rest - Voluntary	4.16	90	5.134	<**.0001**

Bold *p*-values show statistically significant comparisons.

Shifting our focus to attentive behavior to both person and environment, we observed significant differences compared to S1 when the horse interacted with the person, for both levels of familiarity. However, the type of interaction, i.e., S3 vs. S2, did not influence the horse’s attention toward the person or the environment.

### Horse-human causal interactions

The dynamic causal interaction between horses and humans was evaluated by applying time-varying bivariate autoregressive models to the HRV time series of each pair of horses and humans. From those models, we derived the Directed Coherence (DC,^33^) to obtain a measure of causal interaction in the time-frequency domain. Such an index allowed us to observe the interaction in the two main frequency bands characterizing HRV dynamics, namely, the low-frequency (LF, 0.04–0.15 Hz) and the high-frequency (HF, 0.15–0.4 Hz) bands. The HRV dynamics in these two bands are regulated by different autonomic mechanisms. HF primarily reflects parasympathetic control of cardiovascular activity, while LF reflects a complex and less discernible combination of sympathetic and parasympathetic activity, as discussed in.^34^ Accordingly, it is reasonable to assume that the same physiological processes also regulate the DC interactions occurring within these frequency bands. Specifically, the parasympathetic branch predominantly influences the DC interactions in the HF band, while a combination of sympathetic and parasympathetic influences the DC interactions in the LF band.

**Figure 2:**
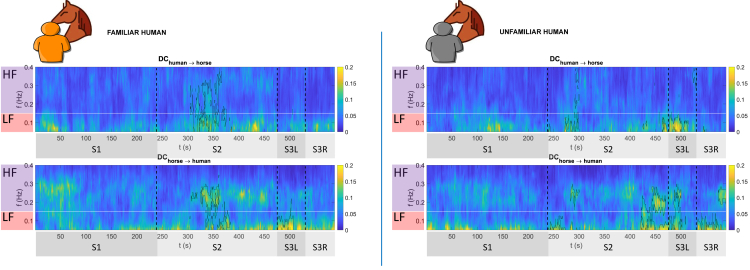
Group-level Directed Coherence (DC) between horses and humans Left: DC between horse and familiar humans. Right: DC between horse and unfamiliar humans. For each direction of interaction (i.e., horse → human and human → horse) a time-frequency matrix is reported. Each matrix has time on the x axis and frequency on the y axis and reports the value of DC for each time-frequency point (t,f). The experimental sessions (i.e., S1: no interaction, S2: free interaction, S3: human-driven interaction) are separated by a vertical black dashed line. LF (0.05–0.15)Hz and HF (0.15–0.4)Hz frequency bands of HRV are highlighted with a horizontal white line and on the left of each DC matrix. Clusters of significant interactions are bounded with a black perimeter for each DC matrix (p−value<0.05, cluster correction).

In Figure 2, we report the time-frequency matrix representation of both $DC_{familiar}$ and $DC_{unfamiliar}$ for both horse → human and human → horse interactions. Each matrix corresponds to a distinct interaction direction (i.e., human → horse and horse → human) for each level of familiarity (i.e., familiar or unfamiliar). These matrices report the directed interactions at the group level, along with their statistical significance. Particularly, they show, for each time-point in the experimental session and across each frequency bin in the range of 0.05–0.4 Hz, the DC at a group level between horse and human HRV signals. The statistical significance of observed causality is assessed through a two-step procedure involving bootstrap and cluster correction. More specifically, we tested the null hypothesis (H0) of no interactions by comparing DC observed values with the distribution of DC values obtained after causality is destroyed through a causal shuffling approach.^35^ In addition, since this procedure is performed for each time-point and frequency bin in each DC matrix, for a total of (4xtxf) points, we handled multiple hypothesis testing with a cluster correction method. Particularly, we considered as significant only those clusters for which p<0.05 and whose size was greater than the biggest cluster observed during S1. Accordingly, we could identify the time-frequency clusters during S2 and S3 that were significant compared to S1 (i.e., a condition of no interaction; more details of the cluster correction method are available in section HRV horse-human causal interactions). In Figure 2, clusters in which causality was significant (p<0.05) are highlighted with a black perimeter.

When subjects and horses were separated (S1), we assumed and observed no specific pattern of interaction in horse-human causal interactions. When the horse and human subjects were together, with the horse free to explore the stationary human (S2), significant synchronization of their heartbeat dynamics occurred. This synchronization happened earlier and in both directions in the case of high familiarity (Figure 2 left), instead, mostly at the end of the S2 session, and from horse to human in the case of unfamiliar interactions (Figure 2 right). However, some bidirectional interactions were also observed at the beginning of the S2 session. Importantly, this synchronization involved both the LF and HF frequency bands. When the horses were groomed by the human subject (S3), the causal interactions exhibited remarkable differences between the two familiarity conditions. The familiar interactions occurred solely from the horse to the human in the LF band, and this was observed only during the half S3 session in which grooming took place on the left side of the horse (S3L) (Figure 2 left). In contrast, for the unfamiliar condition, causal interactions were observed in both directions during S3L, and with almost exclusive predominance direction from the horse to the human during the grooming of the right side of the horse (S3R) (Figure 2 right). Moreover, synchronizations from human to horse occurred exclusively in the LF band, while those from horse to human involved both the LF and HF frequency bands.

### Behavioral and causal correlation analysis

To improve the interpretability of our results, we evaluate whether the observed interactions from physiological signals had a behavioral counterpart. Specifically, we investigated whether the DC casual interaction values were correlated with the behavioral measures of the horse estimated from the video analysis. The correlation was estimated through the Spearman correlation coefficient, while the statistical significance of the correlation was assessed with an *ad hoc* randomization procedure to derive the distribution of the correlation coefficient under the null hypothesis (H0) of no correlation between the time series (more details are given in section f).

**Figure 3:**
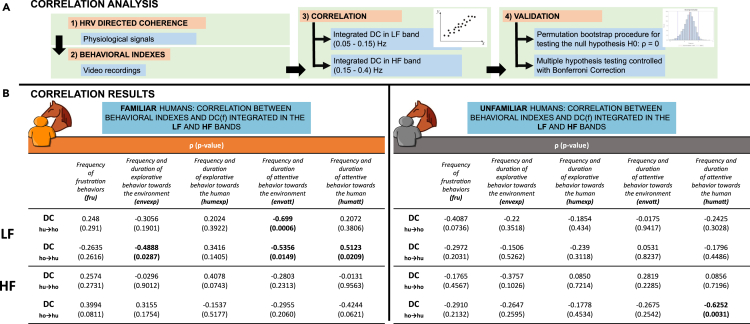
Correlation analysis between DC and behavioral indexes of the horse (A) Correlation is evaluated between the time courses of DC integrated into the LF (0.05–0.15)Hz and HF (0.15–0.4)Hz bands and the time course of each behavioral index. The statistical significance of correlations is assessed using a permutation bootstrap procedure for testing H0:ρ=0. A separate analysis is performed for the familiar and unfamiliar cases. (B) Correlation values along with *p*-values are reported for the familiar (left) and the unfamiliar (right) case. Significant correlations are highlighted in **bold** (p<0.05).

In Figure 3, we report the value of the correlation between DC and behavioral measures along with its associated *p*-value considering the DC integrated in LF and HF bands, respectively. Particularly, we could identify for both LF and HF whether the correlation between physiological measures and behavioral measures was significant. Significant correlations (p<0.05) are reported in Figure 3 and highlighted in bold. The results account for both directions of the interaction and both familiar and unfamiliar individuals. In the LF band, we observed significant correlations only in the familiar case (Figure 3B, left panel, section LF). Particularly, a positive correlation was observed between $DC_{horse\to human}$ and ***humatt*** (ρ=0.5123,p=0.0209). Conversely, a negative correlation was observed between $DC_{horse\to human}$ and ***envexp*** (ρ=−0.4888,p=0.0287) and ***envatt*** (ρ=−0.5356,p=0.0149), and between $DC_{human\to horse}$ and ***envatt*** (ρ=−0.699,p=0.0006). The correlation between $DC_{horse\to human}$ and ***envexp*** and ***humatt*** indicated that for higher values of horse → human causal interaction, a decreased number of ***envexp*** acts, and a number of ***humatt*** acts were observed. The negative correlation between $DC_{horse\to human}$ and ***envatt*** (ρ=−0.5356,p=0.0149), indicates that the higher the horse → human interaction, the lower the attention to the environment of the horse. Considering the HF, we found a single negative correlation for the unfamiliar between $DC_{horse\to human}$ and ***humatt*** (ρ=−0.6252,p=0.0031) (Figure 3B, right panel, section HF), indicating that the higher the horse → human interaction the lower the attention of the animal to the person.

## Discussion

Our study breaks new ground by providing evidence of directional causality in physiological signals between individuals of different species, marking a significant leap forward in our understanding of interspecies interactions. By leveraging methods rooted in the concept of Granger Causality, we have been able to pinpoint the direction of interaction, thereby shedding light on the directed influence of one system (the horse’s ANS) on another (the human’s ANS), and vice versa. If we consider that this mutual influence of the physiological activities of horses and humans occurs when the two actors are close to each other and they are having a behavioral interaction, then we could affirm that what happens in the physiological systems is closely related to the behavior of the two actors. In this case, the behavior of one of the two actors involved in the relationship could represent an unconscious communication system that not only drives the reciprocal interaction but also aligns the physiological response of the other actor. This could have relevant evolutionary outcomes. In the social sphere, aligning behavioral and physiological responses with subjects with whom one is having a physical interaction in close proximity could help the two subjects react in a coordinated manner in the face of environmental stimuli outside the interaction. However, these data do not allow us to determine whether it is the development of behavior during the interaction that directly influences the physiological systems, or vice versa.

Our results underscore the significance of the familiarity level in shaping the outcomes of future encounters between two subjects of different species. Existing evidence has already suggested that horses evaluate humans based on their past interactions.^36^ In a behavioral study involving familiar and unfamiliar people, Fureix and colleagues^37^ emphasized how horses’ memories of previous interactions with humans led them to attribute a generally positive or negative significance to humans, which undoubtedly influenced subsequent approaches. However, our study, shifting the focus from behavioral to physiology analysis, introduces complementary aspects in this regard. The causality analysis in our research not only effectively quantifies the existence of a significant interaction between the two physiological systems in question, but it also highlights the directionality of this interaction. By delineating the influencer and the influence within these interactions, our study offers an interesting perspective on the dynamics of these complex phenomena. That seems to directly link the quality of behavioral interaction with variation in autonomic nervous system activities. However, we could also speculate that our recorded phenomena are related to some evolutionary advantages. More specifically we could presume that when two subjects are in close proximity and they are having a behavioral relationship, this relationship does not represent a threat for either of them. In this case, an unconscious communication system that allows the alignment of behaviors and physiology of the two actors involved in the relationship could represent a serious advantage for both against environmental threats/stimuli. Our data suggests that this could happen also among subjects of different species, far in the evolutive plane.

The session (S1), during which the person and the horse were in separate areas, is considered the baseline and used as a control condition to account for any spurious correlations. This approach, together with statistical correction for multiple comparisons, strongly mitigates the risk that any observed correlations or changes in subsequent sessions could be attributed to the specific factors being investigated, rather than environmental or coincidental influences. When the horse is in control of the interaction (S2), we observe that the autonomical connections are noticeably fewer when the horse is interacting with an unfamiliar person. The more significant connections with the unfamiliar person appear to be driven by the horse, and this becomes particularly evident toward the end of the session when the person becomes somewhat “less unfamiliar.” In contrast, the familiar person induces a bidirectional coupling in the middle of the session. Of note, the band of such horse-controlled interaction interests both LF and HF ranges underscoring the intricate dynamics of the interaction involving both autonomic nervous branches and highlighting the role of familiarity and the horse’s actions in shaping these connections. When the person drives the interaction (i.e., grooming session, S3) the situation appears more complex and focused in the LF domain, highlighting the relevance of the familiarity level between the interactants and the novelty of the design. With the familiar person brushing on the left side (S3L), the horse assumes the lead in the physiological direction ($DC_{horse\to human}$). On the other hand, when interacting from the left with an unfamiliar person, the dynamic encompasses a bidirectional effect, with influence flowing reciprocally from human to horse and vice versa, involving both LF and HF bands. In this case, the results appear to be linked to the horses’ prior experiences. Traditionally, horses are primarily handled from their left side, a practice that commences early in their training (e.g., leading from the left, riding from the left, and veterinary injections) and continues throughout their lives.^37^ Consequently, the left side is where horses qualitatively and quantitatively experience most interactions with people, encompassing both positive and negative valence, spanning years. However, the meaning of actions performed by people on the left side might be perceived differently if these actions are performed by a person who is on the left side, but whom the horse knows and with whom it has an assumed positive interaction that lasts over time. Conversely, when a person that the horse does not know, acts on the left side his or her actions, even if not negative *per se*, could still alter the horse’s emotional state, depending on past experiences. In the S3R phase (i.e., when the interaction comes from the right side), when the interaction involves the unfamiliar person the directionality of causal influence of the autonomic nervous system activity goes from horse to human. This could be related to the difference in the side the horses are used to being handled. On the right side, interaction with an unfamiliar person might still be better managed by the horse’s physiological system, which, on that side, should have less experience with physical interactions with humans anyway. The differences in management between the right and left sides by human beings to which horses are subjected in their lives could be an important variable in the behavioral and physiological bond between horses and human beings. Especially when there is physical contact. Our results seem to indicate that as familiarity drops, the increasing complexity of affinity between the two profiles (human/horse) begins to unfold. This may be attributed to the necessity of acquainting themselves with each other as interacting agents and becoming particularly plausible when the encounter involves tactile contact, such as brushing.

Considering the physiological implications of the LF and HF bands, where LF reflects a combination of sympathetic and parasympathetic activity and HF primarily represents parasympathetic activity, our findings suggest a complex scenario. The interaction between horses and humans appears to trigger a reciprocal influence on the dynamics of their ANS. This influence is characterized by a complex activation of both the parasympathetic and sympathetic branches, rather than reflecting a state of relaxation, which is typically associated with the HF band solely. This suggests that the interaction is not merely a relaxing action, but rather a complex enhancement of ANS dynamics with no clear patterns of results, which is also consistent with the studies on human groups.^14^

By combining the results from the analysis of physiological synchronization with behavioral data, it is possible to show that this physiological synchronization is indeed associated with specific behavioral dynamics. However, this is affected by the familiarity level of the interaction and by the ANS dynamics involved. In familiar human-horse interaction, the increase in LF human-guided synchronization ($DC_{human\to horse}$) is associated with a decrease in the horse’s attention to the environment. Similarly, the rise in horse-guided synchronization ($DC_{horse\to human}$) is linked to a reduced focus on environmental attention and exploration by the horse, but it is also accompanied by an increased emphasis on the attention toward the human. This does not happen in the case of an unfamiliar human, where, instead, there is a single significant inverse correlation between the HF $DC_{horse\to human}$ and the level of attention of the horse toward the person: when the cardiovascular synchronization concerns the parasympathetic system (i.e., in the HF band) there is a loss of attention toward the human probably due to a reciprocal relaxation that brings to a state where the attention to the person is less necessary. This was not totally unexpected, in fact, previous literature reports differences in horse behavior based on their familiarity with humans. Horses tend to look more often at a familiar trainer in a neutral condition such as in our protocol,^38^^,^^39^ while they spend more time monitoring an unfamiliar human only during an obedience task.^40^ Thus, it can be hypothesized that the horse’s behavior toward an unfamiliar person is intricate and less easily quantifiable using conventional methods and patterns typical of behavioral analysis conducted through visual inspection. On the other hand, in the case of the familiar interaction, it is worthwhile noting that among all the behavioral patterns considered in our study, horses’ attention significantly matches with physiological coupling. In particular, we found that such a physiological coupling led by both horse and familiar person negatively correlates with the attention of the horse toward the environment (***envatt***, Figure 3B), meaning that when the influence is reciprocal ($DC_{human\to horse}$ and $DC_{horse\to human}$ not different) the horse’s attention is not focused on noticing the visual field around. Moreover, when the interaction is led by the horse ($DC_{horse\to human}$) a positive correlation with the attention toward the familiar person emerges. In this regard attention, defined as “the selective aspect of perception,”^41^ works as the pre-requisite for adaptive responses. Our data suggest that when the casual directionality is guided by the horses ($DC_{human\to horse}$), their attentive behavior is primarily canalized on humans’ states, with the assumed purpose of catching all the information required to handle the interaction.

Another crucial aspect to consider is the central role of the ANS in regulating emotional states.^42^ The dynamics of social interactions are deeply rooted in empathy and emotional contagion, which occur both intra-species^43^ and inter-species, including between humans and animals.^44^ By evaluating interaction at the level of the ANS, our framework could potentially be extended to the clinical context, especially in the rapidly expanding field of Animal Assisted Interventions (AAI) that incorporate horses in treating human pathologies. Equine-assisted activities (EAT) have been proven to enhance social, emotional, and physical functions in patients suffering from anxiety, depression, autism spectrum disorder, multiple sclerosis, and spinal cord injury or to improve balance, coordination, and posture.^9^^,^^10^ Our data could explain the reason for the success of EATs therapy in which the animal side stimulates the human side’s ANS, triggering the influence of physiological causal directionality. Essentially we found that the ANS of horses affects the response of human ANS (and vice versa). Therefore the horse would be not merely the means through which the pathological human subject gets relaxed or distracted, but the apparently uncoded relationship with this animal could have a definite organic, therapeutic-like action directed at stimulating ANS functions. Future research should include patients with the aforementioned pathologies in the study.

### Limitations of the study

It is worthwhile noting that our study is based on a database that presented several challenges during the signal acquisition phase. Ensuring an effective synchronization between monitoring devices was critical, and we employed highly innovative wearable systems for acquiring both human and equine ECG data comfortably and unobtrusively. The dataset comprises a notable forty distinct human-horse pairs, although they derive from a relatively small number of twenty horses and twenty-two human participants, which could be considered a potential limitation (details about study population are reported in Tables 4 and 5). However, it’s important to highlight that the calculation and of DC measures and the assessment of their significance using bootstrapping was conducted at two levels: at the level of individual human-horse pairs and at the group level. This approach mitigated concerns related to sample size. In addition, it is crucial to emphasize that our study overcomes a common limitation seen in prior experiments examining physiological synchronization among humans during social tasks. Indeed, distinguishing whether the synchronization occurs between individuals or between each individual and the shared behavioral task is often challenging. Our study addresses this issue by design. Not only does our protocol avoid simultaneous task performance by both individuals but, as they belong to different species, their responses to the task stimuli are inherently non-equivalent. Consequently, in our investigation, the synchronization between physiological systems is robust. Another consideration is the selection of frequency bands (0.05–0.4 Hz), which were chosen to align with those typically considered in human studies. Horse’s HRV dynamics may show a broader frequency spectrum (0.01–0.6Hz). However, since the horse’s spectrum includes that of humans, we considered that meaningful synchronizations will occur only within the frequency band common to both interacting individuals.

**Table 4:** Human participants

Number of participants	age (m ± se)	sex	role in equestrian world	average time spent with the horse (day/week) -only for familiar-
20	36.8 ± 2.87	10 males 10 females	7 trainers, 6 riders, 2 professional operators in equine services, 3 experts in equine assisted services, 1 veterinarian	4.12

**Table 5:** Horses population

name	sex	Breed	age	discipline	Workload day/week	n° of people normally interacting with the subject for managment
Dado	m	Sardiniaina anglo-arabian	12	horseback riding flatwork	0	2
Didol	m	argentino	23	school horseback riding	5 (3h/day)	6
Friso	m	friesian	8	horseback riding flatwork	0	2
Neve	f	camargue	21	school horseback riding	5 (3h/day)	6
Remy	m	haflinger	14	horseback riding flatwork	0	3
Arabella	f	sella italiano	28	none	0	2
Arramon	m	haflinger	19	school horseback riding flatwork	4 (2h/day)	4
Betta	f	arabian	9	horseback riding flatwork	5 (1h/day)	3
Danilù	m	sella italiano	10	flatwork	2 (1h/day)	2
Dragonhair	m	sella italiano	10	flatwork	2 (1h/day)	2
Ercole	m	friesian	13	horseback riding flatwork	2 (1h/day)	3
Falco	m	maremmano	13	horseback riding flatwork	2 (1h/day)	3
Oliver	m	haflinger	11	flatwork	4 (1h/day)	3
Saif	m	arabian	8	horseback riding flatwork	5 (1h/day)	3
Sunny	f	hannover	21	none	0	2
Erica	f	monterufoli	8	horseback riding flatwork	3 (2h/day)	5
Ilex	m	monterufoli	4	horseback riding flatwork	3 (2h/day)	5
Gelso	m	monterufoli	6	horseback riding flatwork	5 (2h/day)	6
Uga	f	monterufoli	15	horseback riding flatwork	3 (2h/day)	5
Ironia	f	trotter	15	none	0	4

### Conclusion

To conclude, our study demonstrates and measures the mutual causal directionality influence of autonomic nervous system activity in two different and highly social species as they interact with each other, shedding light on the bi-directionality of such causality. Our finding could provide further input on evolutionary outcomes in social interaction between different species. In the social sphere during close physical interaction, aligning behavioral and physiological responses could help the two subjects react in a coordinated manner in the face of environmental stimuli outside the interaction. Although this is a relevant evolutionary advantage recognized within the same species, our data seem to indicate that these rules should be extended also when interaction in close proximity involves subjects of different species. Considering the ever-growing involvement of horses in pet therapy, this physiological phenomenon could explain the bodily and psychological benefits reported in most cases by users of this therapeutic practice, even though a tangible healing outcome is yet to be approved by the medical community. In addition, we open a window into the study of socio-cognitive sciences, leading the way toward the investigation of the “unconscious” level of communication, which involves the vegetative side of living beings, even if they belong to different species, and regulates their emotional relationship. Beyond the field of pet therapy, this methodology extends its utility to fields as diverse as sports and occupational settings where the human-animal partnership plays a pivotal role. Furthermore, as we venture into the future, researchers may expand their focus to encompass other biological systems, such as the brain and the respiratory apparatus. These uncharted territories offer a rich field for study and may provide invaluable insights into the intricate connections between all living beings, paving the way for innovative approaches to health, well-being, and work.

## Resource availability

### Lead contact

Requests for further information and resources should be directed to and will be fulfilled by the lead contact, Alberto Greco (alberto.greco@unipi.it).

### Materials availability

This study did not generate new unique reagents.

### Data and code availability

• All data reported in this article will be shared by the lead contact upon request.
• All original code is available in this article’s supplemental information.
• Any additional information required to reanalyze the data reported in this article is available from the lead contact upon request.

## Acknowledgments

Research partly supported by the European Union - Next Generation EU, in the context of The National Recovery and Resilience Plan, Investment 1.5 Ecosystems of Innovation, Project Tuscany Health Ecosystem (THE), Spoke 3 “Advanced technologies, methods, materials and health analytics” CUP: I53C22000780001. Research partly funded by PNRR - M4C2 - Investimento 1.3, Partenariato Esteso PE00000013 - “FAIR - Future Artificial Intelligence Research” - Spoke 1 “Human-centered AI”, funded by the European Commission under the NextGeneration EU program. Research partly supported by the 10.13039/501100003196Italian Ministry of Health (Project Code RC IZSVe 15/17).

## Author contributions

All authors designed the research. C. S, A. G, A. L, and P. B., collected the data. A. L. C., C. S., and A. G., analyzed the data. A. L. C., C. S., P. B., and A. G. interpreted the results and drafted the article. All authors wrote the article.

## Declaration of interests

Authors declare no competing interests.

## STAR★Methods

### Key resources table

**Table:** 

REAGENT or RESOURCE	SOURCE	IDENTIFIER
**Software and algorithms**		

Matlab (R2022), The Mathworks Inc.	https://www.mathworks.com	

### Experimental model and study participant details

#### Ethical statement

The study was performed under the ethical standards of the Declaration of Helsinki and with the recommendations of the Italian Animal Care Act (Decree Law 26/2014). The Ethical Committee on Animal Experimentation of the Experimental Zooprophylactic Institute of Venice (IZSVe) approved the experimental protocol in each of its parts (i.e., handling procedures, data collection methods, CE IZSVe 07/2020). Human subjects were enrolled voluntarily, and they signed an informed consent statement to take part in the study. They were advised about their rights, data management, and protection in accordance with the Reg. EU N. 679/2016. The horses’ owners gave written consent to the use of their horses in this experiment.

#### Human subjects

We conducted our study by enlisting human volunteers from various equestrian centres between May and September 2019. Recruitment was facilitated through a network of personal contacts, who in turn engaged volunteers from their respective locations and horse-owning circles. A total of 20 participants were enrolled, with an average age of 36.8 ± 2.87 SE years, comprising 10 females and 10 males. Of these, 11 served as familiar individuals and 9 as unfamiliar counterparts. Each familiar participant was paired with an unfamiliar individual of the same sex. All participants were free from any psychiatric or psychological disorders and had prior experience and confidence in handling horses. A summary of human participants is reported in Table 4.

Unfamiliar handlers were selected from individuals who were either present at the study location or unfamiliar with the horse being tested at the time of the study. To control for potential biases, all participants wore standardized attire—specifically, blue jeans and a blue long-sleeved shirt—during the tasks. Additionally, starting one week before the experiment, all participants were required to use odorless, neutral pH products to eliminate the potential bias of familiar body odor recognition.

#### Animal subjects

Twenty horses (7 females) with an average age of 13.4 ± 1.39 SE years old were enrolled in the study. A summary of horses population is reported in Table 5. They were in good health, showing no signs of injuries or abnormal behaviors. Horses involved in professional equestrian sports were excluded from the selection criteria. The choice of stables for participation was based on specific management criteria, which included an assessment of handling protocols and riding activities. These criteria encompassed the primary activities undertaken by each horse, their daily workload, their level of interaction with humans during activities and for general care, their social interactions with other horses, and their feeding routines. The selected horses were primarily engaged in amateur-level riding activities, involving up to 3 hours of ridden or groundwork per day. They were accustomed to interacting with a range of two to six individuals for daily care and with many more for the activities mentioned. Horses that were group-housed in paddocks were considered, provided they had access to individual stalls for short periods as necessary. This approach aimed to prevent potential stress during experimental tasks that might occur if a horse were isolated in a stall away from its social group. All the horses had continuous access to water, and their pasture diets were supplemented with hay. Some horses also received concentrated feed and small portions of vegetables.

### Method details

#### Experimental protocol

The overall experimental procedure is reported in Figure 1. The experimental protocol consisted of an interaction task with three different conditions, each one lasting 5 min, combining a familiar/unfamiliar human handler test with the concomitant recording of horses’ ECGs. The order of interactions with familiar/unfamiliar humans was randomized^24^:

• The first session (S1) was a no-interaction condition, in which the horse and the human were in separate areas. Specifically, the horse was left free to move in its familiar stall (4 × 4 m), while the person was standing in the stable’s service room.
• The second session (S2) was a horse-driven interaction in which the horse and the human were in the same area. Particularly, human subjects moved from the service room to the stall of the horse itself. They entered, without other humans, and stood still near the door while staring at the floor. During this session, the horse was free to move and explore the environment and the subject, thus driving the interaction by deciding whether to approach, sniff, touch, or stay away from the human.
• The third session (S3) consisted of a human-driven interaction in which the subject took a brush previously positioned outside the box and approached the horse to brush it. The grooming session lasted 2.5 minutes on each side (S3L left side and S3R right side) in a randomized order among the subjects. If the horse tried to move, the person had to maintain contact with it to keep on with the grooming procedure. During this session, the subject had control over the interaction, constantly seeking a connection with the animal. The horse could not avoid the interaction.

### Quantification and statistical analysis

#### Behavioral data acquisition and analysis

To assess horses’ behavioral responses towards human subjects and potential expressions of frustration due to the environmental constraints and/or the presence of a stranger in a reduced space, a specific ethogram has been defined based on the literature (see Table 1 for a detailed description). Via VLC (3.0.6 version) with the plugin Jump-to-Time extension, C.S. analyzed 10 h of videos collected with a digital camera (Sony FDR-AX33) during the three conditions with familiar/unfamiliar humans for each of the tested subjects. The frequency (number of times in which the behavior was displayed during a 30-second time window) of all relevant behaviors performed and duration of those patterns whose length was measurable (specifically, explorative, and attentive behaviors). To check for inter-observer agreement and reliability over scoring, 5 randomly selected 2-minute segments of videotapes were assigned to P.B. The agreement coefficient obtained between the two coders was 0.92.

We conducted a thorough statistical analysis for each behavioral feature, following the 3x2 structure of our experimental protocol, which comprised three experimental sessions and two familiarity levels. It’s worth noting that one horse’s video recordings were unavailable due to technical issues or loss. Our analysis employed mixed-effects linear models. In each model, the variable of interest (e.g., ***fru***, ***envexp***, ***humatt***, etc.) served as the dependent variable, with experimental sessions (S1, S2, and S3) and familiarity (familiar vs unfamiliar) included as fixed effects in an interaction model. Random intercepts were calculated for each participant. The hierarchical nature of mixed-effects models enabled us to establish a unique regression line for each human-horse pair for each variable and subsequently compare the slopes of these regression lines between groups. Degrees of freedom for our mixed-effects models were determined using Satterthwaite’s approximation.

The main effect of interest was the differentiation among the three experimental sessions, the two familiarity levels, and the two-way interactions (experimental sessions *x* familiarity levels). This allowed us to discern distinct responses to S1, S2, and S3 within both the familiar and unfamiliar groups. In instances where these interaction effects were deemed statistically significant, we performed a post hoc simple effect analysis to ascertain whether the experimental sessions exhibited significant differences between the two familiarity groups and whether the two groups demonstrated significant distinctions within each experimental session. To maintain control over Type I errors, we applied Tukey’s method for multiple comparison correction in all inferential tests within the simple effects comparisons. For all the statistical comparisons performed, we considered a significance level of α=0.05.

#### Physiological data acquisition

We acquired ECG signals from horses and humans with two wearable systems. For the horse, the ECG acquisition system consisted of a belt purposely designed to not be more intrusive than a saddle or any similar riding equipment.^45^ The human ECG acquisition system was instead a wearable t-shirt developed at the University of Pisa.^46^ ECG signals from the horse and the human were recorded continuously during the 3 sessions at a sampling rate of 250 Hz. Physiological data processing ECG signals from both the horse and the human were analyzed with Kubios (Biosignal Analysis and Medical Imaging Group at the Department of Physics, University of Kuopio, Kuopio, Finland) to extract HRV time series. To this aim, the R peaks were extracted from the QRS complexes using the Pan-Tompkins algorithm. Algorithm-related artifacts were removed by applying the cubic spline interpolation method. Finally, the obtained RR series were interpolated at 4Hz to derive the HRV signals. These signals were used to estimate causal interactions between the horse and the human.

#### HRV horse-human causal interactions

HRV time series from the horse and the human were used to construct multivariate autoregressive (MVAR) models from which horse-human causal interactions were estimated. MVAR models are one of the most widely used methods for characterizing causal interactions from time-series.^35^ These models extend the concept of Granger Causality, whose principle of causality is expressed in terms of temporal precedence and predictability to the multivariate case. Accordingly, if the prediction error of a time series is significantly reduced by including another time series in the regression model, the second time series is said to have a causal influence on the first. Two additional features of MVAR models are particularly attractive for studying the interaction between different physiological systems. The first is related to time-varying (TV) solutions that allow estimating causal interactions dynamically within a task.^47^ The second is the representation of MVAR models in the frequency domain, which allows for an enhanced physiological interpretation of the observed causalities. Indeed, specific frequency bands may have different physiological meanings depending on the considered biological system.^34^

Here, we used a sliding window approach to estimate bivariate autoregressive (BVAR) models (i.e., a special case of the MVAR model with only two time series) in a time-varying fashion. Particularly, we used 60s-long windows and a step of 1s. The window length was purposely chosen to guarantee robust estimation of model parameters.^48^ Then, the window step length was chosen to track changes in causal interactions smoothly within the task. Models were estimated using the SIFT Toolbox in a machine running Matlab (R2022), The Mathworks Inc. For each window, we estimated model coefficients through the Vieira-Morf algorithm. Then, we derived the Directed Coherence (DC,^33^) to obtain a measure of causal interaction in the frequency domain. In particular, given the TV-MVAR model:

(Equation 1) $$x_{t}=\sum_{k=1}^{p}A_{k}\left(t\right)x_{t-k}+\varepsilon \left(t\right)$$

Where $A_{k}\left(t\right)$ is the M x M coefficient of the matrix of the model at lag k and time t, and $\epsilon_{t}$ is a normally distributed error term at time t such that:

(Equation 2) ε(t)∼N(0,σ)

Given

(Equation 3) $$\bar{A}\left(f\right)=I-\sum_{k=1}^{p}A_{k}\left(t\right)e^{-i2\pi fk}=I-A\left(f\right)$$

where A(f) is the spectral representation of the coefficient matrix A(t).

Then, being:

(Equation 4) $$H\left(f\right)={\left[I-A\left(f\right)\right]}^{-1}=\bar{A}{\left(f\right)}^{-1}$$

the transfer matrix associated with the MVAR model, the DC is given by the following relation:

(Equation 5) $$DC_{ij}\left(f,t\right)=\frac{\sigma_{j}H_{ij}\left(f,t\right)}{\sqrt{\sum_{m=1}^{M}\sigma_{m}^{2}{\left|H_{im}\left(f,t\right)\right|}^{2}}}$$

Since this measure is complex-valued, the modulus or the squared modulus is commonly used to measure connectivity in the frequency domain.^49^ For our analyses, the DC was obtained in the (0,2)Hz frequency range. At the end of this procedure, we obtained, for each horse-human couple, a value of causal interaction for each direction (i,j) (i.e., from j → i: horse → human and human → horse), for each frequency (f) and for each time-point (t). Then, based on the familiarity between the horse and the human we derived two group causal interaction matrices by taking the median over subjects of each $DC_{ij}\left(f,t\right)$ value, obtaining a $DC_{ij}^{familiar}\left(f,t\right)$ and a $DC_{ij}^{unfamiliar}\left(f,t\right)$ matrix. The statistical significance of the observed causalities at the group level was estimated with a bootstrap procedure implementing a group-level causal shuffling approach.^35^ Finally, since we performed a test for each (i,j,f,t) point, we controlled for multiple hypothesis testing using a cluster correction approach. Particularly, we used the size of the biggest cluster observed during S1 as a threshold for clusters observed during S2 and S3. Accordingly, we could estimate significant deviations in connectivity estimates compared to the baseline (S1).

#### Behavioral and causal correlation analysis

The DC values were further used to study the correlation between causal interactions and the behavioral measures of the horse estimated from the videos. Particularly, we were interested in evaluating whether the observed interactions from physiological signals had a behavioral counterpart. To this aim, we estimated the Spearman correlation between the $DC_{ij}\left(f,t\right)$ values integrated into LF and HF bands and each of **fru(t)**, **envexp(t)**, **humexp(t)**, **envatt(t)** and **humatt(t)**. Since behavioral measures were estimated every 30s, we matched their time axis by aligning the center of each 30s-long window with the center of the nearest window used for the DC estimation. Because of the sparse nature of the behavioral measures, we developed an *ad hoc* randomization procedure for estimating the statistical significance of the correlation. Particularly, we randomly permuted DC and behavioral measures in time using 30s long time windows and estimated the correlation between these surrogate time series to obtain its value under the null hypothesis (H0) of no correlation between the two measures. Then, we assigned to each observed value of correlation a p-value based on its position in the surrogate distribution.^50^ Finally, since we tested the correlation separately for each behavioral measure, we corrected the obtained p-values with the Bonferroni method for multiple hypothesis testing.

## Supplemental information

Data S1. Original code, related to STAR Methods section
